# The relationship among fear and anxiety of COVID‐19, pregnancy experience, and mental health disorder in pregnant women: A structural equation model

**DOI:** 10.1002/brb3.1835

**Published:** 2020-09-23

**Authors:** Leili Salehi, Mitra Rahimzadeh, Elham Molaei, Hamideh Zaheri, Sara Esmaelzadeh‐Saeieh

**Affiliations:** ^1^ Research Center for Health, Safety and Environment Department of Health Promotion and Education School of Health Alborz University of Medical Sciences Karaj Iran; ^2^ Social Determinants of Health Research Center Alborz University of Medical Sciences Karaj Iran; ^3^ Clinical Research and Development Center of the Kamali Hospital Alborz University of Medical Sciences Karaj Iran

**Keywords:** anxiety, COVID‐19, fear, mental health, pregnancy

## Abstract

**Introduction:**

Coronavirus pandemic causes stress and anxiety for pregnant women worldwide. The present study was conducted for the path analysis of the relationship among fear and anxiety caused by coronavirus, pregnancy experience, and the mental health of pregnant women.

**Methods:**

This cross‐sectional study was conducted on 222 pregnant women who were referred to Kamali Hospital in Alborz province in 2020. The eligible individuals entered the study through convenience sampling, and data were collected using five questionnaires including the Fear of COVID‐19 Scale, the Anxiety of COVID‐19 Scale, the pregnancy experiences Scales, Depression Anxiety Stress scale, and demographic checklist. The obtained data were then analyzed using SPSS‐16 and Amos software.

**Results:**

According to results of the path analysis, the anxiety of COVID‐19 and concerns during pregnancy were variables that were positively and significantly correlated with mental health only through one path, which was direct, and anxiety of COVID‐19 had also the highest positive direct correlation among them (B = 0.32). The next variable was the happiness during pregnancy experiencing, which had a significantly negative and direct correlation with mental health disorder (B = 0.29). Moreover, fear of COVID‐19 through the mediating concerns of pregnancy experiences was shown to have a significant positive relationship with mental health through an indirect path (B = 0.05).

**Conclusion:**

Based on the result of this study, it is necessary to pay more attention to the mental health of pregnant women during a pandemic. In addition, it is recommended to provide a virtual training group to reduce anxiety caused by coronavirus and pregnancy concerns, as well as emphasizing the feeling of enjoying happiness caused by pregnancy experience during a pandemic.

## Graphical abstract

This paper is path analysis method for explain mental health during pregnancy.

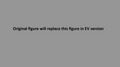

## 1 INTRODUCTION

The prenatal period is often accompanied by maternal mental distress associated with pregnancy itself. Pregnant women are often concern about fetal health and the outcome of childbirth. Besides the anxiety resulted from pregnancy, there are several other risk factors associated with the high anxiety prevalence during pregnancy (Bayrampour, McDonald, & Tough, 2015). Accordingly, one of these factors that can affect the mental health of pregnant women is insecurity related to catastrophic events or natural disasters (Feduniw, Modzelewski, Kwiatkowski, & Kajdy, 2020).

The current COVID‐19 pandemic is considered as an example for a natural disaster with so much global health burden, in which more than 22 million people worldwide are suffering from it and more than 791,000 people died. Correspondingly, Iran has the 11th rank among the countries with the most coronavirus cases in the world, with 350,000 infected people and more than 20,000 deaths (WHO, 2020).

Restrictions related to the social distance that prevents having communication with relatives, friends, and others increase stress, anxiety, and depression in people's daily lives (Mehta et al., 2020).

Pregnant women also face some special challenges due to the responsibility of caring for other children and family members. On the other hand, the need to receive regular care from maternity services increases the risk of exposure to infection with viruses in this population group (Hussein, 2020).

There is no reliable information on pregnancy and its complications during corona infection. However, according to previous epidemics of SARS and MERS, which were associated with several physical and psychological changes during pregnancy (Swartz & Graham, 2020), pregnant women are more affected by the virus. So, coronavirus epidemics cause stress and anxiety among pregnant women in different parts of the world. Concerns and stress during pregnancy would have some side effects such as preeclampsia, depression, the increased nausea and vomiting during pregnancy, preterm delivery, low birth weight, and low Apgar score (Alder, Fink, Bitzer, Hösli, & Holzgreve, 2007; Field et al., 2010; Littleton, Breitkopf, & Berenson, 2007; Qiao, Wang, Li, & Wang, 2012; Rubinchik, Kablinger, & Gardner, 2005).

Physiological changes during pregnancy can also lead to psychological problems and disruption of the socio‐familial roles of women. Consequently, these changes can cause emotional instability and several problems such as stress and anxiety in the mother (Ebadi, Kariman, & Hajifoghaha, 2017).

**FIGURE 1:**
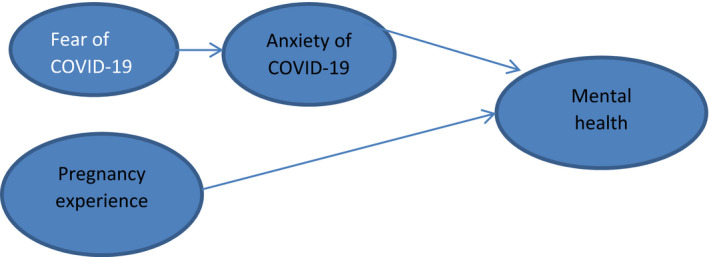
Conceptual model of COVID‐19 pandemic and mental health in pregnant women

The prevalence rate of pregnancy anxiety varies from 15 to 23 percent (Vameghi, Amir Ali Akbari, Sajjadi, Sajedi, & Alavimajd, 2018). Notably, it was found that there is a relationship between pregnancy anxiety and insecurity in pregnant women (Sinesi, Maxwell, O'Carroll, & Cheyne, 2019). Also, mental health is known as an essential factor in maternal health and fetal development (Khatri, Tran, & Fisher, 2019). A study showed that fast‐moving global health crises like COVID‐19 could increase fear and anxiety (Wang et al., 2020). The current COVID‐19 pandemic has caused the increased anxiety among the pregnant women. Since these women are concerned about their unborn child and their own health condition, so COVID‐19 anxiety can also be considered as an influential factor in mental health (Corbett, Milne, Hehir, Lindow, & O’connell, 2020). Researchers observed that pregnancy experiences are affected by fear and anxiety of COVID‐19 in clinical care. However, to date, the relationship between fear and anxiety caused by a coronavirus and pregnancy experience has not been examined, and there is no study on this subject. Therefore, in this study, we sought to assess maternal Fear and anxiety due to COVID‐19, pregnancy experience, and mental health. According to the importance of women's mental health during pregnancy, providing a strategy for early diagnosis and possible interventions in pregnant women affected by pandemics is essential to support psychological adjustment, to prevent the complications of emotional disorders, and to improve prenatal care services. The present study was designed to determine the path analysis fit of the conceptual model (Figure 1), which measures the relationship among fear and anxiety of COVID‐19, pregnancy experience, and the mental health disorder of pregnant women.

## 2 MATERIALS AND METHOD

This cross‐sectional study was conducted during the COVID‐19 pandemic outbreak at Kamali hospital in Alborz province, Iran (during March and April of 2020). This Hospital serves as the main referral center for pregnant women in Alborz. For the purpose of the study, the researcher visited Kamali Hospital, identified the eligible women, and explained the study objectives for them. The study questionnaires were then distributed among the participants. Sampling took place through the convenience method until reaching the determined sample size.

### 2.1 Study population

According to the study by Que et al. (Qu et al., 2012) and considering an alpha coefficient of 0.05, a beta coefficient of 0.2, and a minimum correlation of 0.2 between catastrophic stress and mental health, by applying the formula of $n={\left(Z_{1‐\alpha /2}+Z_{1‐\beta }/0.5ln\left(1‐r/1+r\right)\right)}^{2}+3$, the sample size has been obtained as 194. To increase the accuracy by 10% and considering the potential sample loss, the sample size was estimated as 220 participants.

### 2.2 Inclusion criteria

Iranian woman, aged between 20 and 40 years old, minimum reading and writing literacy, and gestational age higher than 20 weeks in March and April of 2020.

### 2.3 Exclusion criteria

Stressful experiences such as the death of the acquaintance within the past 6 months, addiction, infection by COVID‐19, having any pregnancy complication, and self‐reported psychological or physical illnesses.

### 2.4 Data collection

Data were collected using five questionnaires, including the fear of COVID‐19 Scale (FCV‐19S), Corona Disease Anxiety Scale (CDAS), Depression Anxiety Stress scale (DASS‐21), Pregnancy experience Scale (PES), and a demographic checklist.

### 2.5 Fear of COVID‐19 scale (FCV‐19S)

Fear of the COVID‐19 Scale (FCV‐19S) consists of 7 items measuring the emotional fear reactions toward the COVID‐19 pandemic, and its scoring is on a five‐point Likert‐type scale from 1 to 5. The sum of the scores of these items shows higher level of fear (7–35). Designing and testing validity and reliability of this tool were done by Ahorsu et al. (2020) in Iran in 2020. The scale's Cronbach's alpha was calculated as 0.82.

### 2.6 Corona disease anxiety scale (CDAS)

Corona‐related anxiety is an 18‐item tool that measure corona‐related anxiety in two dimensions, namely psychological symptoms and physical symptoms, and the items are answered on a Likert scale from zero to 3. Each participant receives a score from 0 to 54. The validity and reliability of this questionnaire have been assessed in Iran by Alipour et al. in 2020. Moreover, the Cronbach's alpha for the whole questionnaire was reported (α = 0.919) (Alipour, Ghadami, Alipour, & Abdollahzadeh, 2020).

### 2.7 Depression anxiety stress scale (DASS‐21)

The Depression Anxiety Stress scale was developed by Lovibond & Lovibond in 1995. This questionnaire has 21 items in three dimensions of mental health (depression, anxiety, and stress). Accordingly, each subscale consists of seven items, which are measured using a 4‐point Likert scale (“Never” = 0 point to “Almost Always” = 3 points). In Iran, this scale was psychometric by Asghari, Saed, and Dibajnia (2008), and the Cronbach alpha for the total score of the questionnaire was reported (0.94). Also, the Cronbach alpha for Depression, Anxiety, and Stress scales was 0.85, 0.85, and 0.87, respectively (Asghari et al., 2008).

### 2.8 Pregnancy experiences scale‐brief version (PES‐Brief)

The Pregnancy Experiences Scale‐Brief Version is a 20‐item tool that measures happiness and concerns of pregnancy in two dimensions (each one contains 10 items), and these items are rated on a Likert scale from 0 (not at all) to 3 (a great deal). Ebadi et al. (2017) determined the reliability of this test, total Cronbach's alpha coefficient was obtained as 0.714, and intra‐class correlation was 0.721, which both showed an appropriate reliability of the Persian version tool (Ebadi et al., 2017).

### 2.9 Ethical consideration

An ethics code was obtained from the Ethics Committee of Alborz University of Medical Sciences (IR.ABZUMS.REC.1399.028). The written informed consent was then obtained from the included participants. Also, they were assured regarding their information confidentiality. Moreover, all the spiritual and financial rights of mothers were respected.

### 2.10 Data analysis

This study examined the fit of a conceptual model of path analysis (Figure 1) to determine the Concurrent relationship of fear of COVID‐19, the anxiety of COVID‐19, mental health during pregnancy, and pregnancy experience (concerns and happiness). Therefore, at first, the normality of the quantitative variables was assessed using the skewness and kurtosis test. The significant correlation between variables was analyzed using Pearson's correlation coefficient, and the path analysis was also expressed as a beta. Data were analyzed using AMOS and SPSS‐16 software.

## 3 RESULT

**FIGURE 2:**
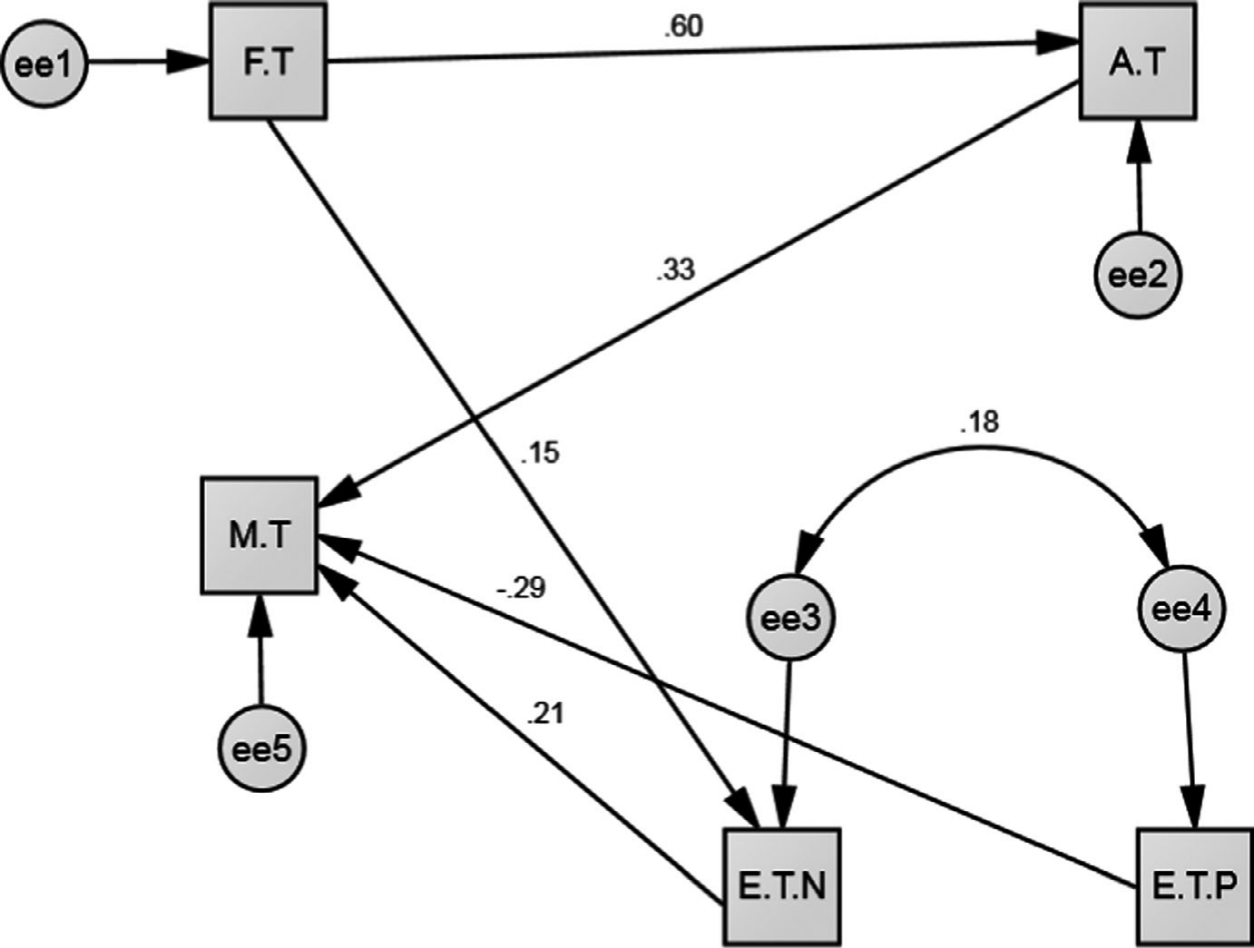
Full Empirical Model (Empirical Path Model between fear of COVID‐19, Anxiety of COVID‐19, pregnancy experiences, and mental health in pregnant women. AT, Anxiety of COVID‐19; ET.N, Pregnancy experience (concerns); ET.P, Pregnancy experience (happiness); FT, Fear of COVID‐19; MT, Mental health

Demographic characteristics of pregnant women are mentioned in (Table 1). According to the results of Pearson's correlation test, mental health during pregnancy was significantly correlated with Fear of COVID‐19, Anxiety of COVID‐19, and happiness and concerns during pregnancy. Among these variables, Anxiety of COVID‐19 had the most significant positive correlation with mental health (*r* = .362, *p* < .001) (Table 2). Based on the results of the path analysis (Figure 2), the Anxiety of COVID‐19 and concerns during pregnancy were those variables that had significantly positive correlations with mental disorder only through one path, which was direct, and Anxiety of COVID‐19 had the highest positive direct correlation among them (B = 0.32). The next variable was the happiness of pregnancy experience, which had a significant, direct, and negative correlation with mental health disorder (B = −0.29). Moreover, fear of COVID‐19 through the mediating concerns of pregnancy experiences also had a significant positive relationship with mental health through the indirect path (B = 0.05) (Table 3).

**Table 1:** Demographic character of Participants

Variable (quantitative)	(Mean ± *SD*)	Minimum	Maximum
Age (year)	29.1 ± 5.6	18	41
Fear of COVID‐19	22.5 ± 5.9	7	35
Anxiety of COVID‐19	15.45 ± 6.03	0	54
Mental health	13.3 ± 8.2	0	40
Concerns pregnancy	16.2 ± 11.9	0	30
Happiness pregnancy	22.8 ± 7.9	0	30

Variable (qualitative)	*F* (%)
Education	
Elementary	31(14)
High School	65(29.3)
Diploma	87(39.2)
University	39(17.5)
Economic	
Inadequate	63(28.4)
Adequate	152(68.5)
Good	7(3.2)
History of abortion	
Yes	19(8.6)
No	203(91.4)
Corona family history	
Yes	5(2.3)
No	217(97.7)
Number of children	
Zero	70(31.5)
1	86(38.7)
2	53(23.9)
>3	13(5.9)
History of disease	
No	169(76.1)
Blood pressure	9(4.1)
Diabetes	16(7.2)
Thyroid	38(17.1)
Cardiovascular	3(1.4)
Other	8(3.6)

**Table 2:** Correlation between Fear and anxiety of COVID‐19, Pregnancy experience, and mental health in pregnant women

	1	2	3	4	5
1. Fear of COVID‐19 (FT)	1				
2. Anxiety of COVID‐19 (AT)	0.60**	1			
	<0.001				
3. Pregnancy experience (happiness) (ET.P)	−0.006	−0.1	1		
	0.93	0.88			
4. Pregnancy experience (concerns) (ET.N)	0.154*	0.179**	0.178**	1	
	<0.02	<0.008	0.008		
5. Mental health (MT)	0.203**	0.362**	0.255**	0.210**	1
	<0.002	<0.001	<0.001	0.002	

** *p* < .001.

* *p* < .05.

**Table 3:** Path Coefficients for study Predictors on mental health in pregnant women

Predictors	Effects		
	Direct	Indirect	Total
Anxiety of COVID‐19(AT)	0.325		0.325
Fear of COVID‐19 (FT)		0.051	0.051
Pregnancy experience (happiness) (ET.P)	−0.29		−0.29
Pregnancy experience (concerns) (ET.N)	0.21		0.21

The results of the model fit indices showed the favorableness and high fit of the model as well as the rationality of the regulated relationships between the variables based on the conceptual model (Figure 2). Accordingly, it was shown that fear of COVID‐19 has a direct effect on the anxiety of COVID 19 in the primary model, due to the confirmation of the effect of fear on pregnancy concerns, and we added this relationship to the final model (Corbett et al., 2020). The result of the goodness‐of‐fit index showed the effect of fear on concerns during pregnancy. The results of the primary and final model are mentioned in (Table 4).

**Table 4:** Goodness‐of‐fit indices for the Model

Fitting index	*χ* ^2^	*df*	X2/*df*	*p*	CFI	GFI	NFI	RMSEA
Primary model	8.364	4	2.09	.079	0.973	0.985	0.952	0.07
Final model	3.08	4	0.77	.54	1	0.99	0.98	0.00
Acceptable range			X2/*df* < 5	>.05	Larger than 0.90 is an acceptable model		Larger than 0.90 is an acceptable model	The range between approximately 0 and 0.1 is an acceptable model

Abbreviations: CFI, Comparative Fit Index; GFI, Goodness‐of‐fit statistic; NFI, Normed‐fit index; RMSEA: Root mean square error of approximation; *χ* ^2^: chi‐square.

## 4 DISCUSSION

The results of this study show that anxiety caused by coronavirus had a direct impact, which was mostly related to mental health disorders during pregnancy.

Some studies have shown that the greatest desire in healthcare providers in mental health is focusing on depression; however, pregnancy anxiety is an important disease leading to harmful outcomes during pregnancy. COVID‐19 pandemic also had a global impact on the mental health of individuals (Ahorsu et al., 2020).

The results of a study showed that at the time of suffering from COVID‐19, people mostly showed negative emotions (anxiety, stress, and depression) compared to positive emotions and took more care of themselves with negative emotions (Fakari & Simbar, 2020).

Other studies have also shown that, during public health emergencies like SARS, several negative emotional responses such as high levels of anxiety have been occurred (Li, Wang, Xue, Zhao, & Zhu, 2020).

The results of the present study show that fear of corona during pregnancy could affect the anxiety caused by corona as well as indirectly affecting the mental health during this period by affecting the concerns of pregnancy.

One of the main causes that increase anxiety during the pandemic period is the fear of COVID‐19, and one of the most common of which is the fear of infecting others or infecting beloved ones with the disease (Colizzi et al., 2020).

The results of some previous studies have shown that women usually are more afraid of COVID‐19 than men, and these high levels of fear in women can be due to the gender differences in sensitivity and susceptibility to stress, and the increased risk of mental health problems following the occur of stressful life events (Bitan et al., 2020; Limcaoco, Mateos, Fernandez, & Roncero, 2020; Maunder et al., 2003).

During the coronavirus pandemic, pregnant women feel fear from their illness and their family members when commuting to hospitals, and because of these stress and anxiety, they want to terminate their pregnancies prematurely or by cesarean. These pregnant women feel fear and anxiety on the health of their fetus and baby during the pandemic period (Ahorsu et al., 2020).

The results of the present study show that pregnancy concern as the second variable was directly related to maternal mental health disorders during this period.

At the end of pregnancy period, women experience high levels of anxiety and signs of fear about the unpredictability of childbirth (Qiu et al., 2020). Psychological changes are common during pregnancy and many factors are involved in its increasing or decreasing, which not only causes women to consider pregnancy as a cause of happiness, but also as a cause for concern (Sheen & Slade, 2018). During pregnancy, mothers suffering from infectious diseases are more concerned about their baby's health (Mahboubeh, Abbas, & Nourossadat, 2016). In one study, repeated use of disinfectants during pregnancy to fight the COVID 19 virus was considered as a cause of concern in the pregnant woman because of the risk to the fetus (Fakari & Simbar, 2020).

The occurrence of stressful events during pregnancy, such as the death of relatives, exposure to natural disasters or the occurrence of a pandemic that cause interpersonal imbalances, lack of contact with relatives, occupational problems, etc., could increase the concerns during pregnancy (Holditch‐Davis et al., 2015). A systematic review study found that, although stress, life stress events, and exposure to stressors during pregnancy are associated with the increased risk of mental health problems and are considered as mental health problems, these data are not approved in many studies (Graignic‐Philippe, Dayan, Chokron, Jacquet, & Tordjman, 2014).

The results of the present study show that happiness caused by pregnancy experience has a direct inverse effect on mental health disorder during pregnancy, which is a factor that increases its experience during pregnancy and also reduces the problems of maternal mental health during this period.

The results of a previous study showed that the occurrence of COVID‐19 pandemic initially caused negative emotions in people, but positive emotions were then increased, which were caused by the group cohesion of people in belief and prayer compared to the conditions created (Li et al., 2020).

In this study, the feeling of happiness from pregnancy was a hope source of for mothers, and by more emphasizing on this experience, mental health can be improved compared to the conditions created.

According to the vulnerability of pregnant mothers in the pandemic period and their susceptibility to infection, it is recommended to pay special attention to their fears and to identify the mental disorders of women during this period.

Therefore, according to the direct effect of anxiety caused by coronavirus on the mental health disorder of pregnant mothers during the pandemic period, it is recommended to conduct a qualitative study on identifying the causes of maternal anxiety during this period.

One of the limitations of this study was the lack of measuring fear and anxiety of fathers during the pregnancy of their wives and the amount of support for wife in the pandemic period, which is considered as an important and influential factor on maternal mental health, so it is recommended to conduct more studies in this field.

Another limitation of the study was the type of study. In this regard, in some studies, the cross‐sectional method has been mentioned to be weak in explaining the conditions needed for testing mediation; however, due to the pandemic conditions of the COVID‐19 and the stress and anxiety of pregnant women, we decided to design a study with less time consumption. Therefore, it is recommended to perform more prospective studies to investigate the effect of fear and anxiety of the coronavirus on the mental health and pregnancy experience of mothers.

## 5 CONCLUSION

The results of this study show that anxiety caused by corona directly and the fear caused by corona indirectly and by affecting pregnancy worries are related to the mental health of pregnant women. So, providing information on increasing maternal awareness of coronavirus, its risk factors, and its impact on the fetus and baby is essential. Also, according to the effect of happiness caused by pregnancy experience on mental health during this period, and recommending to reduce the number of care during this period and commuting restrictions in healthcare centers, it is necessary to provide group virtual training for reducing anxiety caused by corona and pregnancy concerns. Also, the feeling of enjoying happiness caused by pregnancy experience during a pandemic should be emphasized.

## CONFLICT OF INTEREST

No conflict of interest.

## AUTHOR CONTRIBUTIONS

Sara Esmaelzadeh is a supervisor who designed and performed the study; Leili Salehi designed and consulted about the proposal and sampling; Mitra Rahimzadeh is a biostatistician and gave consultation on sampling and analysis of data; Elham Molaei performed sampling; and Hamideh Zaheri performed the study and sampling.

### Peer Review

The peer review history for this article is available at https://publons.com/publon/10.1002/brb3.1835.

## Data Availability

The data that support the findings of this study are available in the supplementary material of this article.
